# Ex vivo assessment of the buccal and oral bone by CBCT

**DOI:** 10.1007/s00056-021-00335-w

**Published:** 2021-08-09

**Authors:** M. Ruetters, T.-S. Kim, D. Hagenfeld, D. Kronsteiner, H. Gehrig, C.-J. Lux, S. Sen

**Affiliations:** 1 Section of Periodontology, Department of Operative Dentistry, University Hospital Heidelberg, Im Neuenheimer Feld 400, 69120 Heidelberg, Germany; 2 Section of Endodontology, Department of Operative Dentistry, University Hospital Heidelberg, Im Neuenheimer Feld 400, 69120 Heidelberg, Germany; 3 Department of Orthodontics, University Hospital Heidelberg, Im Neuenheimer Feld 400, 69120 Heidelberg, Germany; 4 Institute of Medical Biometry and Informatics, University Hospital Heidelberg, Im Neuenheimer Feld 130.3, 69120 Heidelberg, Germany; 5 Department of Periodontology and Restorative Dentistry, University Hospital Münster, Waldeyer Str. 30, 48149 Münster, Germany

**Keywords:** Cone beam computed tomography, Computed radiography, Periodontitis, Periodontal bone defects, Orthodontics, Digitale Computertomographie, Computerradiographie, Parodontitis, Parodontale Knochendefekte, Kieferorthopädie

## Abstract

**Purpose:**

Identifying buccal and oral bone as an important supporting periodontal structure for teeth provides important information for treatment planning in periodontics and orthodontics. This study aims to add evidence to the knowledge of preciseness of cone beam computed tomography (CBCT) measurements of the vertical dimension of buccal and oral bone. The hypothesis is that CBCT is an accurate and reliable method to measure vertical vestibular and oral bone loss.

**Methods:**

The amount of vertical buccal and oral bone loss (bl) of 260 sites of 10 human cadavers was investigated clinically and radiographically by CBCT. Radiographic measurements were rated by two blinded raters. Measurements and the corresponding differences between clinical and radiological findings are described by medians and quartiles (Q1–Q3). For statistical analysis, Lin’s concordance correlation coefficient (CCC) and Bland–Altman plots were calculated.

**Results:**

The CCC between the raters was 0.994 (95% confidence interval 0.992–0.995). The median bone loss (bl) distance from the cementoenamel junction (CEJ) to the bony defect (BD) was 3.5 mm (range 3–5 mm). The median bl measured in the CBCT was 3.8 mm (range 3.1–4.8 mm). The median difference of the 2 measurements for all sites included in the study (*N* = 260) was −0.2 mm (−0.7 to 0.3 mm).

**Conclusions:**

CBCT seems to be an accurate and highly reliable method to detect and describe vertical buccal and oral bone loss. It could improve planning and prediction for successful combined periodontal and orthodontic therapies.

## Abstract (de)

**Zielsetzung:**

Die Identifizierung von bukkalen und oralen Knochen als wichtige unterstützende parodontale Struktur für Zähne liefert wesentliche Informationen für die Behandlungsplanung in der Parodontologie und Kieferorthopädie. Ziel der Studie ist es, das Wissen über die Genauigkeit von CBCT(„cone beam computed tomography“)-/DVT(digitale Volumentomographie)-Messungen der vertikalen Dimension von bukkalem und oralem Knochen zu erweitern. Die Hypothese ist, dass die DVT eine genaue, zuverlässige Methode zur Messung des vertikalen vestibulären und oralen Knochenverlustes ist.

**Methoden:**

Das Ausmaß des vertikalen bukkalen und oralen Knochenverlusts (bl) an 260 Stellen von 10 menschlichen Leichnamen wurde klinisch und röntgenologisch mittels CBCT untersucht. Die röntgenologischen Messungen wurden von 2 verblindeten Gutachtern bewertet. Die Messwerte und die entsprechenden Unterschiede zwischen klinischen und radiologischen Befunden werden durch Mediane und Quartile (Q1-Q3) beschrieben. Für die statistische Analyse wurden Lins Konkordanzkorrelationskoeffizient (CCC) und Bland-Altman-Plots berechnet.

**Ergebnisse:**

Der CCC zwischen den Ratern betrug 0,994 (95%-Konfidenzintervall 0,992–0,995). Der mediane Knochenverlust (bl) von der Schmelz-Zement-Grenze (CEJ) bis zum Knochendefekt (BD) betrug 3,5 mm (Bereich 3–5 mm). Der in der DVT gemessene mediane bl betrug 3,8 mm (Bereich 3,1–4,8 mm). Die mediane Differenz der beiden Messungen für alle in die Studie einbezogenen Stellen (*N* = 260) betrug −0,2 mm (−0,7 bis 0,3 mm).

**Schlussfolgerungen:**

Die DVT scheint eine genaue und sehr zuverlässige Methode zur Erkennung und Beschreibung von bukkalem und oralem vertikalen Knochenverlust zu sein. Sie könnte die Planung und Vorhersage für erfolgreiche kombinierte parodontale und kieferorthopädische Therapien verbessern.

## Introduction

Identifying the amount of buccal and lingual bone as a periodontal structure supporting teeth provides important information for treatment planning in several fields of dentistry such as periodontics and orthodontics. Diagnostic radiographic techniques, frequently used in daily orthodontic practice, are lateral cephalometric radiographs and the panoramic view [1, 2]. Both methods are helpful diagnostic tools that provide a two-dimensional image of the bone anatomy [2, 3]. Unfortunately, none of these methods shows the buccal or oral aspect of bone at a single tooth. The lateral cephalometric radiograph only shows a superposition of the front teeth and their oral and buccal bone [2]. For instance, orthodontists may use information of this periodontal structure for deciding their treatment modality. The role of bone covering the lower incisors is an issue often discussed among orthodontists [4]. These teeth experience gingival recessions, maybe caused by excessive movement—naturally or iatrogenic—out of the bone [5]. Recessions can also be diminished by orthodontic treatment [6]. Several studies investigating the correlation of orthodontic health conditions and the amount of buccal bone present around teeth have been published. However, these results vary. Casanova-Sarmiento et al. analyzed mandibular anterior alveolar thickness and height in individuals with different sagittal and vertical skeletal relationships [7]. They concluded that there is no influence on the alveolar thickness or height, even if dental compensation was present, due to sagittal skeletal relationship. An earlier study by Raber et al. concluded that the thickness of labial alveolar bone over the incisors varied based on the underlying skeletal discrepancy in each patient. According to this study skeletal discrepancy influenced the inclination of the maxillary and mandibular incisors [8].

If the dimension of buccal bone is known before treatment, fenestrations followed by gingival recessions may be prevented by reducing the amount of planned tooth movements. Several imaging techniques have already been analyzed with respect to their capability to visualize the buccal bone lamina. Cone beam computed tomography (CBCT) is one of these techniques. There are already studies using this technique as a method to investigate the correlation between the amount of bone and the position of the lower incisors, as aforementioned [7, 8]. However, only a few studies, with small sample sizes, have dealt with the precision of CBCT in detecting buccal and oral bone or its loss [9, 10]. One study identified CBCT as a method that might overestimate the true clinical situation [9]. Another study ascertained that even high-resolution CBCT could not reliably depict the thin buccal alveolar bone covering. Moreover, this study also concluded that there was a risk of overestimating fenestrations and dehiscences [10].

More studies are needed to verify whether CBCT is indeed a suitable technique for this periodontal–orthodontic issue. One limitation of CBCT studies is that use of different devices makes them difficult to compare due to different properties of the devices used and their capability to illustrate particular anatomic markers or structures [11, 12]. This study aims to add more evidence to the knowledge of the precision of CBCT measurements of buccal and oral bone by using a Sirona Galileos device (Dentsply Sirona, Bensheim, Germany).

We assert that CBCT is an accurate and reliable method to measure vertical vestibular and oral bone loss.

## Materials and methods

### Clinical examinations

**Fig. 1:**
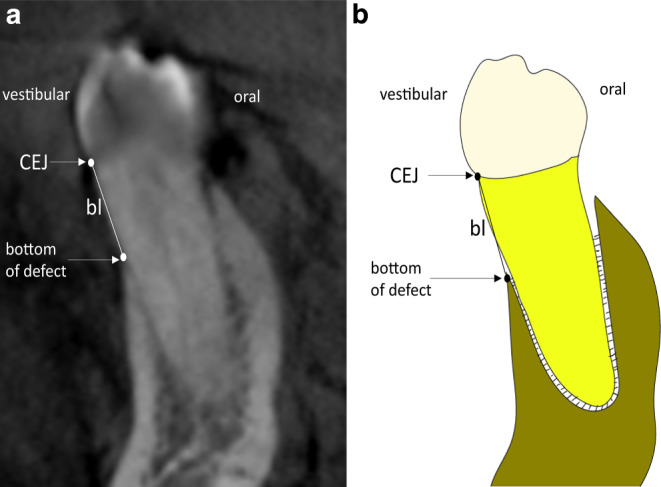
CBCT of tooth 35 and schematic drawing of teeth 35 for clinical measurements: **a** oral and vestibular measurements (bl) of a representative tooth in CBCT in coronal view, clinical measurement of tooth 35, **b** schematic drawing for clinical measurements. *bl* distance from cementoenamel junction (CEJ) to bottom of defect DVT des Zahns 35 und schematische Zeichnung des Zahns 35 für klinische Messungen: **a** orale und vestibuläre Messungen (bl) eines repräsentativen Zahns in der DVT in koronaler Ansicht, klinische Messung des Zahns 35, **b** schematische Zeichnung für klinische Messungen. *bl *Abstand zwischen Schmelz-Zement-Grenze (CEJ) und Boden des Defekts

For this ex vivo study, 20 half-sectioned heads of 10 human cadavers with a total amount of 292 oral and buccal sites of 146 teeth were investigated clinically and radiographically by CBCT (Sirona Galileos, 98 kV, 30 mAs, scan time: 14 s, field of view: 15.4 × 15.4 × 15.4 cm^3^). The isotropic voxel size was 0.25 mm. The cadavers in this study have already been used in a previous study [13]. The sites and data of this study have not been used in any other study yet. The study was approved by the ethical review board of the University of Heidelberg (S-410/2015). The cadavers were preserved with 99% ethanol, glycerin, and 37% formalin and were placed in the CBCT machine with a tube as a holding. The throat was positioned and fixated in the tube and the chin was fixed at the edge of the tube so that the occlusal plane was parallel to the positioning line. The gingiva was removed after the radiographic examination, in order to ensure a realistic image, and the vertical bone loss was measured with a periodontal probe (a PCP-UNC 15, Hu-Friedy, Chicago, IL, USA) at two sites (buccal and oral) per tooth on 146 teeth. Accordingly, 292 sites were measured. The bone loss (bl) was defined as the distance from the cementoenamel junction (CEJ) to the bottom of the bone defect (BD; Fig. 1). The measurements of bl at the cadavers’ teeth were performed by one calibrated investigator (MR) with a PCP-UNC 15 periodontal probe (Hu-Friedy, Chicago, IL, USA) and are described as “clinical measurements” in the following text [14]. For calibration, the investigator had to reproduce pocket depth measurements successfully with a standardized ex vivo reference model (Co. M. Tech., South Korea) at 168 sites compared to one of the principal investigators (relative agreement of 95%).

### Radiographic examinations

Measurements of the mid-buccal and mid-oral bone level at the spot between the CEJ and the bottom of the defect were performed in the CBCT by two blinded calibrated investigators (MR and SS) with more than 6 years of experience with CBCT diagnostics (Fig. 1). For calibration, they independently measured 150 sites in CBCTs according to the measurements in this study. In exceptional cases, deviations of more than 0.5 mm, measurements were observed and have been discussed until consensus was found.

CBCT images with artefacts were rejected to avoid bias, e.g. those with extinction artefacts, beam hardening artefacts, partial volume effect or aliasing artefacts obscuring the CEJ or the bottom of the defect, which made assessment impossible [15].

**Fig. 2:**
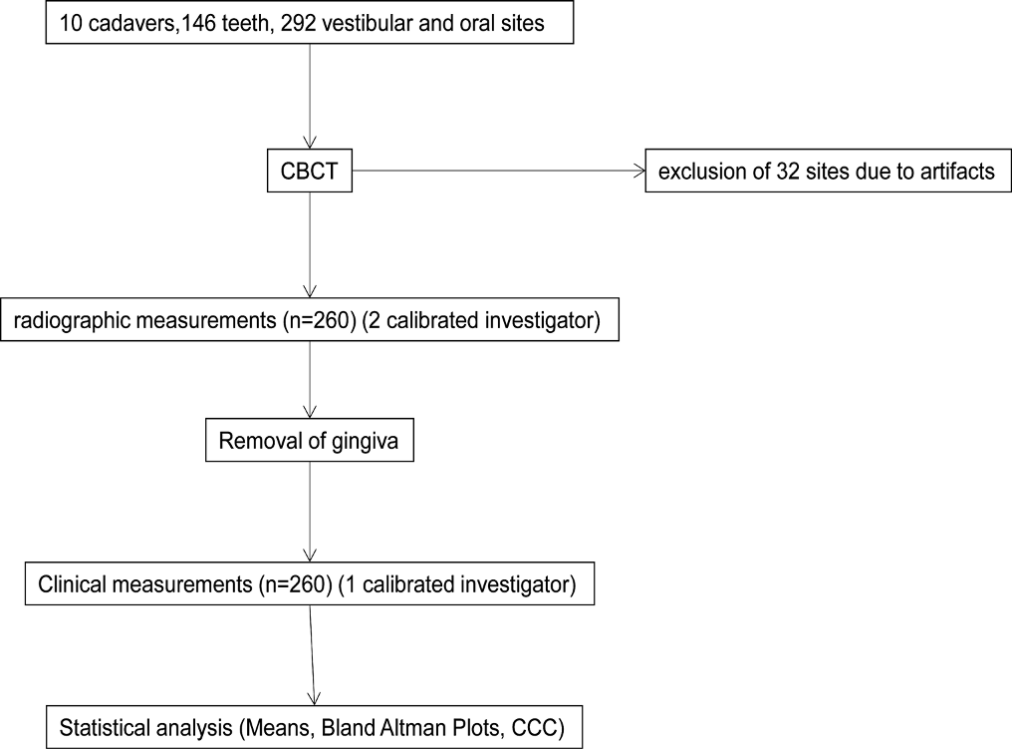
Flow chart of the study. *CBCT* cone beam computed tomography, *CCC* concordance correlation coefficient Ablaufdiagramm der Studie. *CBCT* digitale Volumentomographie, *CCC* Konkordanzkorrelationskoeffizient

The software application used was OSIRIX pro (aycanOsiriX 2.06.000, aycan Digitalsysteme GmbH, Würzburg, Germany). Windowing and levelling were allowed. Measurements were all performed on a certified monitor (RadiForce RS 210, EIZO, Hakusan, Japan) in the same dark room. A flow chart of the study is shown in Fig. 2.

### Statistics

Measurements were descriptively documented by median and quartiles (Q1–Q3). Differences between radiographic and clinical measurements were calculated by the subtraction of radiographic measurements from clinical measurements. Bland–Altmann plots were created including 95% limits of agreement where the clinical measurements were defined as gold standard to detect possible over- or underestimation and systematic bias. Lin’s concordance correlation coefficient (CCC) was calculated to assess the interrater reliability [16]. For statistical analysis the software R version 4.0.2 in combination with the packages “BlandAltmanLeh” and, “DescTools” were used [17–20].

## Results

After exclusion of 32 sites due to artefacts, 260 sites were investigated radiographically in CBCT and compared to the clinical measurements. Of these measurements, 109 (41.9%) were in the upper jaw, and 151 (58.1%) were in the lower jaw. The median of the clinically evaluated distance CEJ-BD (bl) was 3.5 mm (3–5 mm) the median of bl in found in CBCT was 3.8 mm (3.1–4.8 mm).

The CCC between rater one and rater two was 0.994 (95% confidence interval [CI] 0.992–0.995).

The median for the difference of the 2 measurements for all sites included in the study (*N* = 260) was −0.2 mm (−0.7 to 0.3 mm). The median for the teeth in the upper jaw was −0.1 mm (−0.6 to 0.2 mm), and that for the teeth in the lower jaw was −0.2 mm (−0.8 to 0.3 mm).

### Front teeth

Looking especially at the front teeth measurements in the upper jaw (*N* = 53), the median of the differences was −0.3 mm (−0.7 to 0.1 mm). In the lower jaw (*N* = 58), sites showed a median of the differences of −0.5 mm (−1 to 0.2 mm).

When comparing buccal and oral sites, the following results could be seen: measurements at the buccal sites of upper front teeth (*N* = 27) showed a median of differences of −0.3 mm (−0.7 to 0.1 mm) compared to −0.3 mm (−0.8 to 0 mm) at the oral sites (*N* = 26). Lower front teeth showed a median of the differences of −0.5 mm (−1 to 0.1 mm) at the buccal sites (*N* = 27) versus −0.6 mm (−1 to 0.4) at the oral sites (*N* = 31).

### Premolars and molars

Sites at the premolars and molars in the upper jaw (*N* = 56), showed a median of the differences of −0.1 mm (−0.4 to 0.3 mm). In the lower jaw (*N* = 93), the median of the differences for these teeth was −0.1 mm (−0.6 to 0.5 mm).

When comparing buccal and oral sites, premolars and molars in the upper jaw showed a median of the differences of −0.1 mm (−0.3 to 0.3 mm) at the buccal sites (*N* = 28) and −0.1 mm (−0.7 to 0.3 mm) at the oral sites (*N* = 28). In the lower jaw, the median of the differences was −0.1 mm (−0.8 to 0.2 mm) at the buccal sites (*N* = 46) compared to −0.1 mm (−0.5 to 0.6 mm) at the oral sites (*N* = 47).

The medians and corresponding quartiles (Q1–Q3) are shown in Table 1.

**Table 1:** Clinical measurements, cone beam computed tomography (CBCT) measurements and resulting bone loss (bl) differences Klinische Messungen, DVT(digitale Volumentomographie)-Messungen und daraus resultierende Unterschiede im Knochenverlust (bl)

	*N*	Median bl clinical (mm)	(Q1–Q3)	Median bl CBCT (mm)	(Q1–Q3)	Median (bl clinical–bl CBCT) (mm)	(Q1–Q3)
Total	260	3.5	3–5	3.8	3.1–4.8	−0.2	−0.7 to 0.3
Total upper jaw	109	3.5	3–5	3.8	3.0–4.8	−0.1	−0.6 to 0.2
Front upper jaw	53	3.0	2.5–4	3.6	2.8–4.6	−0.3	−0.7 to 0.1
Front upper jaw vestibular	27	3.5	2.5–4	3.6	2.9–4.9	−0.3	−0.7 to 0.1
Front upper jaw oral	26	3.0	2.5–4	3.5	2.8–4.2	−0.3	−0.8 to 0
Posteriors upper jaw	56	4.0	3–5	4.2	3.3–5.2	−0.1	−0.4 to 0.3
Posteriors upper jaw vestibular	28	4.0	3.25–5	4.2	3.2–5	−0.1	−0.3 to 0.3
Posteriors upper jaw oral	28	4.0	3–5.25	4.1	3.3–5.3	−0.1	−07 to 0.3
Total lower jaw	151	3.5	3–5	3.9	3.2–4.8	−0.2	−0.8 to 0.3
Front lower jaw	58	3.0	3–5	3.8	3.4–5.5	−0.5	−1 to 0.2
Front lower jaw vestibular	27	3.5	3–6.5	4.2	3.5–6.7	−0.5	−1 to 0.1
Front lower jaw oral	31	3.0	3–4.5	3.7	3.1–4.4	−0.6	−1 to 0.4
Posteriors lower jaw	93	3.5	3–5	3.9	3.2–4.5	−0.1	−0.6 to 0.5
Posteriors lower jaw vestibular	46	3.25	3–4.5	3.8	3.2–4.9	−0.1	−0.8 to 0.2
Posteriors lower jaw oral	47	4.0	3–5	3.9	3.1–4.3	−0.1	−0.5 to 0.6

*bl* bone loss: distance from cementoenamel junction to bottom of defect

**Fig. 3:**
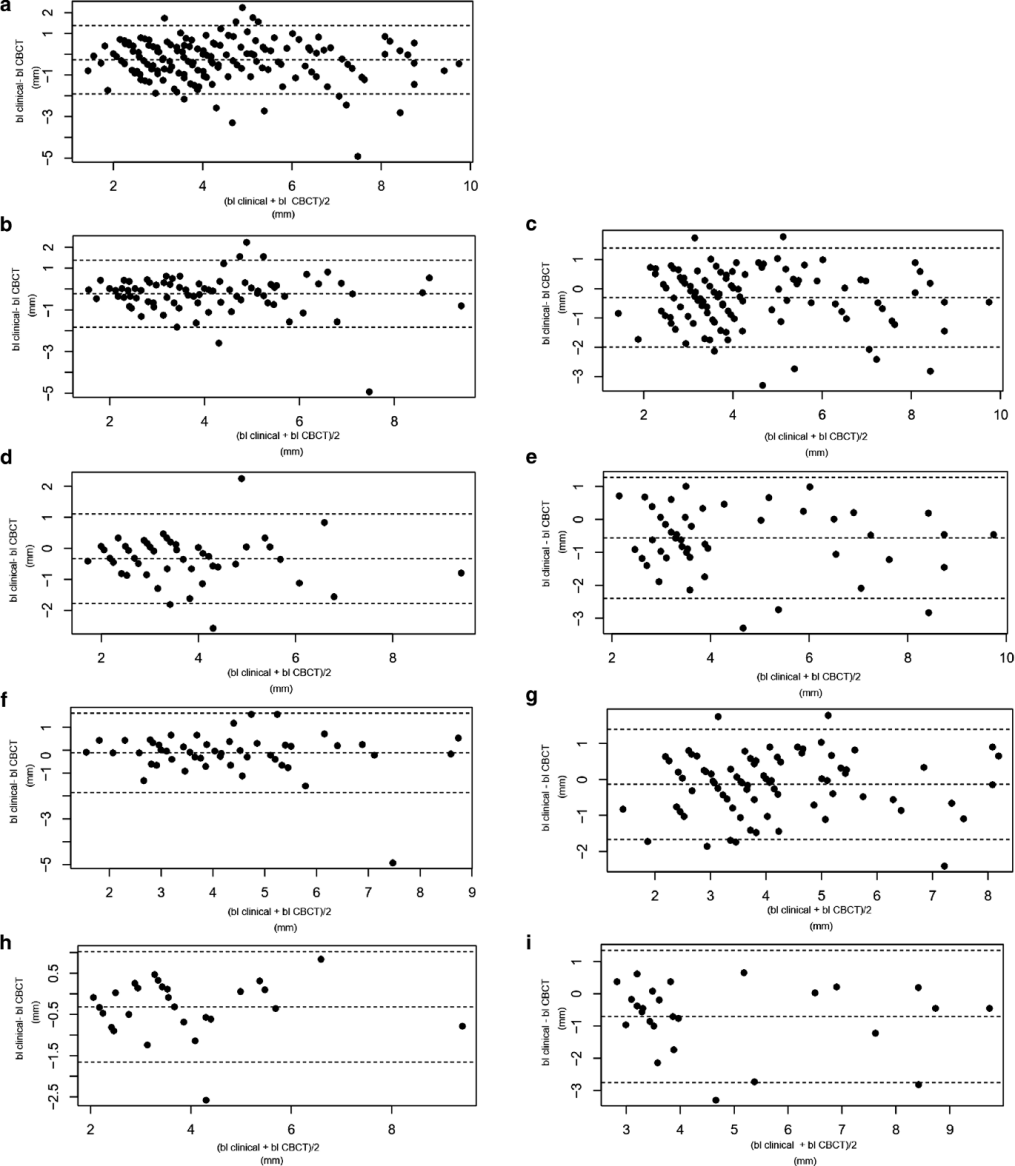
Bland–Altman plots: **a** all measurements, **b** all measurements at upper jaw, **c** all measurements at lower jaw, **d** all front teeth upper jaw, **e** all front teeth lower jaw, **f** all posteriors upper jaw, **g** all posteriors lower jaw, **h** all vestibular measurements front teeth upper jaw, **i** all vestibular measurements front teeth lower jaw. *Upper* and *lower dashed lines* 95% of values are in between these two lines, *middle dashed line* mean difference. No systematic bias can be seen Bland-Altman-Plots: **a** alle Messungen, **b** alle Messungen am Oberkiefer, **c** alle Messungen am Unterkiefer, **d** alle Frontzähne im Oberkiefer, **e** alle Frontzähne im Unterkiefer, **f** alle Backenzähne im Oberkiefer, **g** alle Backenzähne im Unterkiefer, **h** alle vestibulären Messungen an den Frontzähnen im Oberkiefer, **i** alle vestibulären Messungen an den Frontzähnen im Unterkiefer. *Obere und untere gestrichelte Linien* 95% der Werte liegen zwischen diesen beiden Linien, *mittlere gestrichelte Linie* mittlere Differenz. Ein systematischer Bias ist nicht zu erkennen

The Bland–Altman plots (Fig. 3) show the mean of the differences. The 95% limits of agreement range of the differences each time between clinical measurements and measurements in CBCT can be seen for different locations represented by the highest and lowest lines (Fig. 3). No obvious signs of systematic bias can be observed.

## Discussion

The hypothesis of this study was that CBCT is a reliable method to measure linear periodontal buccal and oral bone loss and it can be confirmed for the CBCT machine used. The high CCC (0.994) indicates excellent interrater reliability.

Neither location of the tooth (upper versus lower jaw, front versus side teeth), nor location of the defect (oral or buccal), nor the type of tooth had an impact on the measurements’ precision. CBCT showed high concordance with the clinical measurements. The Bland–Altman plots illustrate a very similar range for the 95% of agreements at all locations, also indicating that the precision was independent of the tooth location. This is in line with other studies that have analyzed the mesial and distal aspects of alveolar bone [13]. Overall, CBCT seems to slightly overestimate the buccal and oral bl. The highest overestimation for bl was seen at vestibular sites of the lower incisors. The results are in line with existing studies [9, 10]. However, those studies only investigated mandibular incisors. The present study examined premolars and molars as well as upper incisors, at buccal as well as at oral sites.

The instrument used in this study was also different from previous studies. While the other authors used a KaVo 3D exam (KaVo, Biberach, Germany), the present study was conducted with a Sirona Galileos device (Sirona Galileos, 98 kV, 30 mAs, scan time: 14 s, field of view: 15.4 × 15.4 × 15.4 cm^3^). The isotropic voxel size in our study was 0.25 mm. Patcas et al. used two different voxel sizes, 0.125 mm and 0.4 mm. They showed similar results as our study (0.6 mm/0.3 mm vs 0.7 mm ± 1.05 SD). The overall tendency for the differences is the same in both studies. CBCT overestimates the bl. The slightly higher differences found in our study might be caused by the clinical probe used being less detailed. Patcas et al. used a digital calliper with an accuracy of 0.01 mm [10], whereas the accuracy in the current study was 0.5 mm.

In comparing the results within our study, no clear difference could be observed between the different locations and tooth types. The buccal aspects in the lower front teeth had the highest difference in measurements, most likely due to the fact that the horizontal bone thickness in this area is usually the thinnest compared with other regions. The isotropic voxel size of 0.25 mm means that the machine cannot illustrate thinner areas.

With respect to the ALADA (as low as diagnostically acceptable) principle, CBCT has a higher radiation dose compared to other imaging methods. According to the FDI policy statement, all reasonable means should be used to reduce radiation exposures, without compromising diagnosis [21]. In orthodontics, a lateral cephalometric radiograph and the panoramic view are required for an initial diagnosis. The effective radiation dose of a panoramic view is typically between 4.7 and 14.9 mSv [22], and that of a lateral cephalometric view is about 5.6 mSv [23]. This amounts to 10.3–20.5 mSv per patient, depending on the machines used. For CBCT machines, a review by Ludlow et al. estimated effective doses of 13–769 mSv for large or medium FOVs, and 7–521 mSv for small FOVs depending on the machine and the acquisition protocols used [12]. This can lead to a similar effective dose for a CBCT and the combination of a panoramic and a lateral cephalometric view, depending on the machine and the protocol used. If all necessary information for an adequate orthodontic treatment can be acquired with a low-dose CBCT, this technique would be a suitable replacement for the two standard diagnostic methods. Accordingly, only one method could yield more information, leading to safer treatment and possibly avoiding undesirable periodontal conditions such as mucogingival recessions [5].

Limitations of this study must be mentioned for completeness. This study only includes data sets with a large field of view of one machine, which resulted in a higher amount of radiation exposure. Newer generations of CBCTs include low-dose protocols and the possibility of smaller fields of view [24]. Future studies should investigate these protocols with respect to their capability of imaging buccal and oral periodontal bone loss. However, if data sets of the machine used in this study already exist, one can use these to judge the presence of buccal and oral bone.

The accuracy of the clinical measurements was 0.5 mm. The accuracy of CBCT measurements was assessed to the nearest 0.01 mm. This must be taken into consideration when interpreting the results of this study and the potential overestimation of bone loss if using CBCT. Clinically relevant orders of magnitude are about 1 mm. In reference to the Bland–Altman plots, most of the differences between clinical and radiographic measurements were within 1 mm, indicating that most of the sites could be appropriately analyzed as presented in the clinical situation.

Another limitation is the character of this study. Owing to the fact that this was ex vivo, no motion artefacts deteriorated the quality of the images. In daily clinical routine, motion artefacts are one of the problems that may minimize the information on CBCTs by reducing the local resolution of the images [15].

## Conclusion

Within the limitations of this ex vivo study, CBCT seems to be an accurate method to detect and describe vertical periodontal buccal and oral bone loss. It could help to improve treatment planning and predict the biological limitations of the alveolar envelope for orthodontic therapies. The use of CBCT imaging to view periodontal structures and conditions for orthodontic reasons must be carefully justified, and further studies evaluating low dosage protocols are required.
